# Re-introduction of vivax malaria in a temperate area (Moscow region, Russia): a geographic investigation

**DOI:** 10.1186/s12936-020-03187-8

**Published:** 2020-03-18

**Authors:** Varvara A. Mironova, Natalia V. Shartova, Andrei E. Beljaev, Mikhail I. Varentsov, Fedor I. Korennoy, Mikhail Y. Grishchenko

**Affiliations:** 1 Faculty of Geography, Lomonosov Moscow State University, Moscow, 119991 Russia; 2 WHO Global Malaria Programme, Geneva, Switzerland; 3 A.M, Obukhov Institute of Atmospheric Physics, 3 Pyzhyovskiy Pereulok, Moscow, 119017 Russia; 4 Research Computing Center, Lomonosov Moscow State University, Moscow, 119991 Russia; 5 FGBI Federal Center for Animal Health (FGBI ARRIAH), Vladimir, 600901 Russia; 6 Faculty of Geography and Geoinformatics, Higher School of Economics, Moscow, 101000 Russia

**Keywords:** Vivax malaria, Autochthonous cases, Re-introduction, Modelling, Geospatial analysis, Environmental determinants, Climate favourability

## Abstract

**Background:**

Between 1999 and 2008 Russia experienced a flare-up of transmission of vivax malaria following its massive importation with more than 500 autochthonous cases in European Russia, the Moscow region being the most affected. The outbreak waned soon after a decrease in importation in mid-2000s and strengthening the control measures. Compared with other post-eradication epidemics in Europe this one was unprecedented by its extension and duration.

**Methods:**

The aim of this study is to identify geographical determinants of transmission. The degree of favourability of climate for vivax malaria was assessed by measuring the sum of effective temperatures and duration of season of effective infectivity using data from 22 weather stations. For geospatial analysis, the locations of each of 405 autochthonous cases detected in Moscow region have been ascertained. A MaxEnt method was used for modelling the territorial differentiation of Moscow region according to the suitability of infection re-emergence based on the statistically valid relationships between the distribution of autochthonous cases and environmental and climatic factors.

**Results:**

In 1999–2004, in the beginning of the outbreak, meteorological conditions were extremely favourable for malaria in 1999, 2001 and 2002, especially within the borders of the city of Moscow and its immediate surroundings. The greatest number of cases occurred at the northwestern periphery of the city and in the adjoining rural areas. A significant role was played by rural construction activities attracting migrant labour, vegetation density and landscape division. A cut-off altitude of 200 m was observed, though the factor of altitude did not play a significant role at lower altitudes. Most likely, the urban heat island additionally amplified malaria re-introduction.

**Conclusion:**

The malariogenic potential in relation to vivax malaria was high in Moscow region, albeit heterogeneous. It is in Moscow that the most favourable conditions exist for vivax malaria re-introduction in the case of a renewed importation. This recent event of large-scale re-introduction of vivax malaria in a temperate area can serve as a case study for further research.

## Background

*Plasmodium vivax* is one of the species of human malaria that is evolutionary well adapted to temperate climatic conditions. Its agent has lower temperature requirements in its extrinsic cycle than other human malaria species. The presence of hypnozoites that are responsible for its long latency allows the parasite to survive seasons when air temperatures prohibit malaria transmission [1]. Vivax malaria was widespread during the period of its greatest presence in Europe (19th Century), occurring even in the North (in southern England [2–4], southern Sweden [4], and being a serious problem in Finland [5]).

There is very little information on malaria in Russia before the mid-19th century [6]. Favr [7] observed that “the incidence of malaria in Russia should be considered at least 5 million cases per year; it takes first place among all the diseases of the Russian people”. After the Bolshevik Revolution of 1917, the incidence of malaria in the USSR, formerly Imperial Russia, showed repeated ups and downs while remaining quite high. The highest level of malaria cases was recorded in 1934 (about 10 million cases) [8]. At that time, *P. vivax* largely predominated in temperate areas of European Russia and Siberia, whereas *Plasmodium falciparum* was widespread in sub-tropical Central Asia and Transcaucasia, which did not belong to Russia proper. The limit of transmission of vivax malaria corresponded roughly to the border of the southern and middle taiga; it was endemic in the southern parts of Arkhangelsk region and occasionally it reached the city of Arkhangelsk itself (64º N) [7].

A misconception exists among non-specialists, which is shared and fostered by mass media, by which the northern limit of malaria is attributable to the limit of the vector’s distribution. This is not the case since *Anopheles* mosquito populations are much less demanding of heat and may thrive in areas where the development of *P. vivax* in mosquitoes is impossible due to the shortness of the warm season.

Malaria was widespread in the Moscow region in the first half of the 20th Century, especially in its central parts and in the east, where large-scale peat extraction was under way at that time. It attracted migrants from neighbouring provinces and created numerous anopheline breeding sites [9]. Areas of peat production accounted for up to 50% of the total number of malaria cases in the Moscow region [10]. The cessation of peat mining significantly improved the situation in its eastern part, although malaria transmission persisted in the region until the early 1950s when it was interrupted.

In the course of the global malaria eradication campaign in mid-20th Century, the disease was eliminated on the European continent. The term “Europe” is used in a strictly geographic sense throughout this text i.e. the western part of Eurasia which is limited by the Urals mountains, Ural river and the Great Caucasian chain. This is to be distinguished from the WHO European Region, which includes some areas that do not belong to Europe proper, such as Asian Turkey, Asiatic Russia, the countries of the Caucasus and Central Asia, Israel and Cyprus. However, at the beginning of the 21st Century, several countries in the WHO European Region faced re-introduction and even re-establishment of malaria, most of them belonging to former Soviet republics. Malaria re-emergence was observed in 9 countries by 2000 [11]. Greece, which had been free from malaria since 1974, experienced a malaria recurrence in 2010–2013 [12]. In those cases, only vivax malaria restarted transmission, whereas falciparum malaria, which is a species most often imported into Europe from the tropics, was not transmitted by local vectors, probably because of the incompatibility of Afrotropical and Oriental *P. falciparum* with Palearctic mosquitoes [13].

Russia has been exposed to large-scale importation of vivax malaria since the dissolution of the USSR in 1991. The greatest challenge was importation from Tajikistan, the country that faced a malaria epidemic in the post-Soviet era. The peak of malaria cases in Tajikistan was officially registered as nearly 30,000 in 1997 [14], although the true number of cases, according to expert estimates, could have exceeded 100,000 per year [15].

**Fig. 1:**
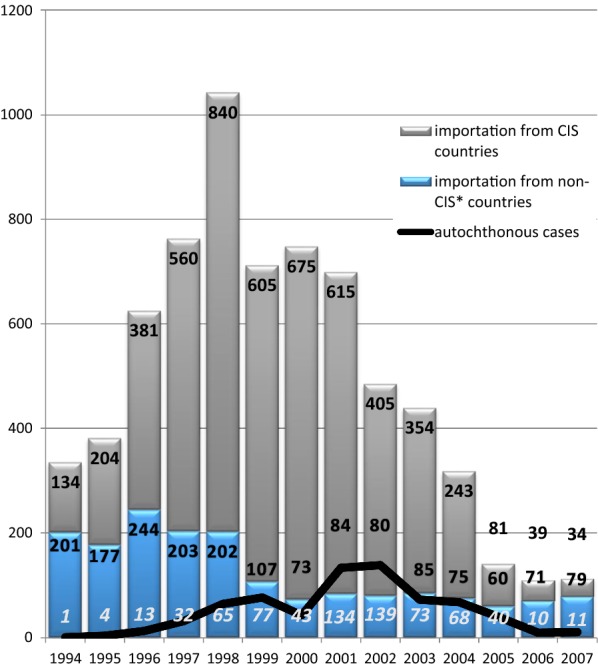
The dynamics of imported and autochthonous cases in Russia, 1994–2007

The dynamics of imported cases is presented in Fig. 1. By origin, the cases have been split into two groups, viz cases from the “near abroad” (newly independent states of the ex-USSR) and cases from the “far abroad” (all other countries). Imported cases from the “near abroad” were always due to *P. vivax,* whereas *P. falciparum* and other species were present in the latter group. The importation reached its peak in 1998 and fell gradually afterwards. This process accelerated in 2005 due to an improvement of the situation in the donor countries [14]. The curve of autochthonous cases in Russia mirrored that of the cases imported from the “near abroad” with a lag of 3 to 4 years.

Due to an increase in the influx of refugees and labour migrants from ex-USSR republics and a weakening of epidemiological control, cases of autochthonous malaria began to emerge in various areas of Russia (mainly in the European area). During the period from 1995 to 2008, 525 autochthonous malaria cases were registered in the European part of Russia [16]. The most difficult situation arose in the Moscow region, which accounted for about half of all recorded autochthonous malaria cases. A sharp deterioration in the situation occurred in 2001 when transmission of malaria resumed not only in rural communities of Moscow region, but also in Moscow city itself, which is exceptional for temperate latitudes. Vivax malaria transmission in the region occurred every year until 2008, although a few sporadic cases were observed later, too.

It should be noted that malaria transmission in temperate and sub-tropical zones of Europe is not rare. After malaria elimination in 1950s, cases of transmission were reported in Corsica [17–19], Italy [20], Spain [21], Bulgaria [22], and Greece [12, 23, 24]. Cases of transmission were observed in rural communities and small towns almost exclusively. The situation in Russia was significantly worse than these outbreaks, in terms of duration, number of cases, territorial extent and in involvement of large cities.

The post-elimination history of malaria in Europe indicates that Russia, and Moscow region in particular, are especially prone to re-introduction of vivax malaria. The situation in Moscow region once again demonstrated that this disease is able to re-emerge quite easily in those areas where it was widespread in the past. The aim of the study is to present this large-scale malaria re-introduction in Moscow region at the beginning of the 21st Century (1999–2008) to the scientific community and to analyse the geographical determinants of this outbreak. There was no intention to produce a full epidemiological description of the event.

## Methods

### Study area

Moscow region includes two administrative units of the Russian Federation that have considerable autonomy: the city of Moscow and Moscow Oblast. The border between them was changed in 2012, but this text refers to the pre-reform status. At the beginning of 2012, the population of Moscow and Moscow Oblast was 11.6 million and 7.2 million, respectively, and the areas were 2561.5 sq km and 44,329 sq km, respectively (data of Russian Federal State Statistics Service) [25]. The region is in the centre of the East European Plain, at altitudes from 97 to 310 m above sea level. It is characterized by presence of various types of settlements ranging from metropolitan areas of Moscow to small villages. The region is highly developed economically and attracts large numbers of labour migrants, primarily from the countries of Central Asia.

The climate of the region is temperate continental and sufficiently wet (average temperature is –10 ℃ in January and + 19 ℃ in July, average annual precipitation is 713 mm). Over the past decades, Moscow region was subject to significant climatic changes and is characterized by spatial heterogeneity of the thermal regime. A specific feature of urban climate is the presence of an urban heat island (UHI) causing substantial difference in the temperatures of the city and the suburban or rural areas [26]. On average, the centre of Moscow megacity is 2 ℃ warmer than surrounding rural areas [27], however, the urban–rural temperature difference could reach up to 14 ℃ [28]. According to the recent observational and modelling studies, the urban-induced temperature anomaly covers the whole city and its nearest suburbs, beyond its administrative limits [29, 30]. Moreover, there has been an intensification of the UHI of Moscow, which is caused by urban growth and development and is especially pronounced in summer [27, 31]. Moscow region has a dense and extensive network of rivers and streams, numerous lakes with a total area of more than 130 sq km, as well as many ponds. Many of these water bodies are suitable for Anopheles breeding. In general, natural conditions for existence of both vectors and the pathogen (*P. vivax*) are favourable throughout the region.

### Entomological and epidemiological data

Fauna, distribution, abundance, and phenology of vectors of malaria in Moscow region were studied in detail in the 1950–60s. Unfortunately, updated entomological information is scanty, due primarily to lack of interest in malaria in the 1990s. The observations, however, continued uninterrupted, albeit patchy, and they did not demonstrate any major change in vector bionomics or in proxy indicators (such as anophelogenic surfaces). At least four species of *Anopheles* are present in Moscow region, including three belonging to the complex *of Anopheles maculipennis s.l*. (*Anopheles maculipennis*, *Anopheles messeae*, *Anopheles beklemishevi*) and *Anopheles claviger*. Of these, *An. messeae* is believed to play a central role in malaria transmission. The key epidemiological role belongs to females of the first and partly second generation hatched in May–June [32–35].

In Central Russia, mosquitoes of *Anopheles maculipennis* complex breed usually along the banks of moderate-sized water bodies (ponds, lakes) with shallow, warm and clean water. Semi-aquatic vegetation (reeds) is essential. Larvae may also thrive around floating islets of duckweed. They never breed in small containers, like neglected pots or barrels. In general, they rarely breed within the households, unless small dugouts exist for decorative purposes. Due to generally enough water supply for domestic needs, owners do not make dugouts for storing water.

As a rule, every rural settlement has at least one pond or small lake serving for firefighting and recreational purposes, which are perfect breeding places for anophelines. In cities and towns breeding places do exist, mostly in park areas, but their anophelogenic productivity is not high, due to industrial and domestic pollution, periodic removal of aquatic vegetation, streamlining banks and larviciding in some of them.

Due to the lack of updated information on vectors, an entomological factor has not been included in this study. Accordingly, this study is based on official records of Rospotrebnadzor, the organization responsible for surveillance of infectious diseases in Russian Federation. All autochthonous cases of vivax malaria have been parasitologically confirmed and epidemiologically investigated. Autochthonous cases are those cases that occur due to a transmission through local vectors. They are the aggregate of (i) introduced cases that originate immediately from an imported case and, (ii) indigenous cases that originate from any other case due to transmission by mosquitoes within a given area [36]. Cases of vivax malaria occur either early (10–14 days after infection), or later, mostly within the next transmission season (usually 9–12 months after the inoculation). They are denoted as short and long incubation cases, respectively. However, they are not distinguished in official records. Relapses along with primary cases are being counted as one case, i.e. only once. In total, 405 autochthonous cases of vivax malaria recorded in Moscow and Moscow Oblast from 1999 to 2008 have been analysed. Cases have been mapped using ArcGIS software.

### Climate and environmental data

The choice of indicators for analysing the factors that influenced the occurrence and the distribution of cases during the recent re-introduction of malaria in the region relates to the following considerations:

- the impetus to malaria transmission was provided by a large-scale importation from countries of the former USSR affected by malaria epidemics amid favourable meteorological conditions in the recipient regions;
- spatial heterogeneity of the distribution of cases caused by several environmental factors.

To analyse the degree of climatic favourability for development of sporozoites, observational data from Moscow weather stations was used in the period from the start to the peak of the outbreak (1999–2004). The stations are in the city centre (Balchug), city parks (VDNKh and MSU) and other urban areas (4 stations), as well as 15 stations located in Moscow Oblast.

The integrated database of continuous (8 times a day) meteorological observations was created using the archives of the Russian Institute for Hydrometeorological Information—World Data Centre (RIHMI-WDC), Central Department for Hydrometeorology and Environmental Monitoring, Meteorological Observatory of Lomonosov Moscow State University.

To analyse the territorial heterogeneity of the distribution of cases the following variables of climate and environmental data that were available in continuous spatial resolution were used (Table 1). All variables were presented in the form of rasters, cropped according to the shape of the territory (Moscow region), reduced to a total resolution of 1 × 1 km and converted to ASCII format. The selection of these particular climatic variables was guided by long-established consensus that malaria is associated with summer temperatures in the most predictable way [43, 44]. Precipitation has a strong influence on malaria, which has long been well-known [43, 45], however its linkage to malaria is not linear. All depends on the breeding habits of the local vectors. Altitude and vegetation density have been referred to by the same authors as important factors of malaria.

**Table 1:** Climate and environmental variables

Variable	Description and data source
Climate data	
Maximum temperature of the warmest month	Gridded data, average for 1970–2000 with a spatial resolution 1 × 1 km [37, 38]
Annual precipitation	
Natural environmental data	
Altitude above sea level	Digital terrain model ASTER DEM with a spatial resolution of 30 m
Vegetation density	The maximum green vegetation fraction [39, 40]
Landscape division	Regional vectorized landscape map [41]
Man-made environmental data	
Building density	Open street map data
Density of roads	
Density of railways	
Distance to railway stations	
Density of cottage communities	Vectorized map of cottage communities’ locations [42]
Distance to cottage communities	

A popular WorldClim gridded data set with 1-km spatial resolution were used [38] as a source of climatic information for geospatial analysis. It is emphasized that despite high spatial resolution, the WorldClim data on air temperature is not perfect, especially for urban areas, where significant underestimation of temperature spatial variability was revealed [46]. WorldClim data reasonably resolves the elevation-induced local climate features, e.g., lower daytime temperatures in the lowlands, however the representation of Moscow UHI was found to be unsatisfactory. The decision to use WorldClim data on daily maximum temperatures is driven by a reason that they are less affected by urban-induced effects in comparison to daily-mean and nocturnal temperatures. WorldClim data allow accounting for regional-scale temperature gradients, small-scale orography-induced effects, but not for urban climate features.

As postulated by Beklemishev [47], each landscape unit has its own combination of physiographic and ecological features that determines a particular quality of that unit vis-à-vis malaria. Therefore, the landscape grid is tantamount to a malariological stratification grid.

The landscape stratification of the Moscow region is well developed [41] and provided with appropriate cartographic material. The borders between the landscape units are usually well distinguishable even for an observer in the field.

Building density is used as an indirect indicator of human population and development. The density of roads and railways, as well as the distance to railway stations, are used as indicators of the presence and intensity of mobility of the population. The density of the so-called ‘cottage communities’ and the distance to them are used to identify links with possible sources of infection.

Cottage communities are a new type of human settlements in Russia that proliferated around big cities in the 1990s. Typically, cottage communities consist of a few dozen two-story standardized buildings with a small plot of land attached and are located 20–50 km from the border of Moscow. Each house is usually occupied by one or two families. Their occupants belong to the wealthy segment of Moscow city dwellers who usually work in Moscow and own apartments in the megacity. The developers preferred to use migrant labour for the construction works, mostly from Tajikistan, which experienced large-scale epidemics of malaria at that time.

Water bodies are numerous in Moscow region, especially near rural settlements. However, not all of them are suitable for anopheline breeding. Since there are no simple means to tell anophelogenic reservoirs from innocuous ones using only remote sensing, the proximity to water bodies could not be considered as one of malaria determinants in this geospatial analysis.

Based on the research concept and the need to solve two independent tasks: to analyse the degree of favourability of climatic conditions for extrinsic development of vivax malaria pathogen at the time of the outbreak and to perform the spatial analysis of the distribution of autochthonous cases, two different methods were used.

### Climate favourability assessment

To assess the degree of favourability of climatic conditions for vivax malaria, the Moshkovsky’s method has been used [48], the method, which was being used in Russia for routine monitoring conditions for extrinsic development of malaria parasites since the 1950s. Moshkovsky adapted to malaria the ideas of Bodenheimer [49], who had developed practical ways to predict timing of development of insects and plants. Information required are average daily temperatures (ADTs) for the period of development of the organism in question (sporozoites in this case). The threshold temperature of development for each particular species is derived from experimental data. The difference between the ADT for each day and the threshold temperature is called an effective temperature. When accumulated effective temperatures measured in degree-days (or the *sum of temperatures*, as it is usually denoted in Russian or French texts) reach a particular level, this heralds the accomplishment of a particular developmental stage of the organism.

For the purpose of malaria, the method has been recommended by the WHO [50, 51]. According to Moshkovsky, extrinsic development of *P. vivax* requires the accumulated sum of 105 degree-days above the threshold of 14.5 ℃. In this study, the sums of effective temperatures (*P. vivax)* have been calculated for each year between the beginning and the peak of the outbreak (1999–2004). As a result, the temporal limits of the following overlapping elements of malaria season have been identified for each year.

- the season of effective temperatures: the part of year during which the ADTs are consistently above the threshold temperature;
- the season of effective infectivity of mosquitoes: the period during which a full development of malaria parasites in mosquitoes (from gametocytes to mature sporozoites) is possible.

### Geospatial analysis

To analyse the spatial heterogeneity of the distribution of cases caused by various environmental factors, the method for modelling ecological niches with optimization based on the maximum entropy principle [52] was applied using MaxEnt software. This instrument performs the selection of the probability distribution of a biological species in question over the study area on the basis of: (a) the known locations in which this species was found (presence data), and, (b) a set of spatial variables characterizing the territory in question. The method is widely used for: (i) modelling the potential range of distribution of a species; and, (ii) modelling the potential area of distribution of the disease based on the assumption of its coincidence with the pathogen’s geographical range. Examples of the latter approach are studies by Peterson et al. and Rose and Wall on viral diseases [53, 54]; Du et al. on myasis [55]; Abdrakhmanov et al. [56, 57]; Mwakapej et al. [58] on anthrax. A conceptual review of the application of the MaxEnt method in biogeography is given in [59]. For malaria, this method was applied in predicting environmentally suitable areas for several *Anopheles* species in Iran [60].

All variables were checked for multicollinearity using the Raster Correlation procedure from the additional SDMtoolbox toolkit for ArcGIS [61]. To determine the possible correlation between all analysed variables and malaria cases, toolkit Band collection statistics analysis in ArcGis software was used.

MaxEnt modelling was carried out using 10 replications, the results of which determined the average values and the confidence interval boundaries of the distribution of the territory suitability for malaria transmission. Assessment of the contribution of each variable in the model reflects a change in its quality when the value of the variable in question changes and when the value of the others is fixed. The weight of the variables for constructing the models was estimated using the jackknife method, based on a comparison of the simulation results with the sequential exclusion of each variable (or when modelling using only one variable).

The predictive ability of the MaxEnt model was estimated by comparing the model’s ability to correctly predict the presence and absence points using the area under the curve indicator (AUC) [62], which represents the area under the receiver operating characteristic (ROC). Since the MaxEnt model is based on the presence data only, randomly generated points (pseudo-absent points) are used as the absence data. The ROC curve shows the relationship between the fraction of presence data correctly predicted by the model (sensitivity) and the proportion of the incorrectly predicted pseudo-absence data (1-specificity). The AUC value lies between 0.5 and 1.0 where 0.5 denotes a bad classifier and 1.0 denotes an excellent classifier [63].

In order to obtain the best model complexity, a regularization coefficient is used that modulates the fit of the model and allows reducing over fitting. The higher values of the coefficient provide simpler models resulting in broader areas predicted to be suitable for the species under study [64]. Eight regularization coefficient values from 0.5 to 4.0 in 0.5 steps were tested. The best value was chosen based on the highest AUC provided.

## Results

### Spatial distribution of malaria cases

**Fig. 2:**
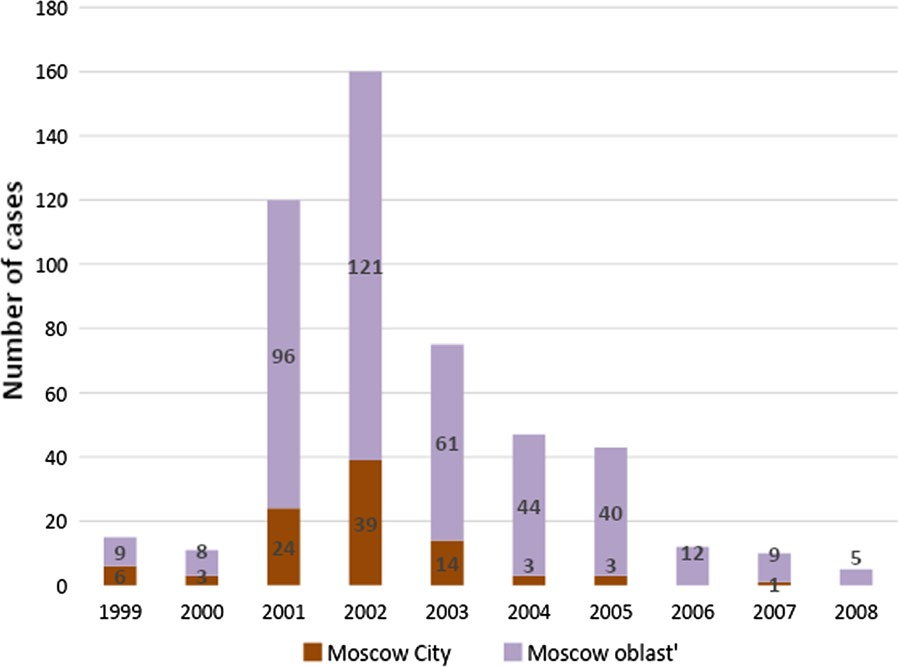
Autochthonous cases of vivax malaria in Moscow region, 1999–2008

**Fig. 3:**
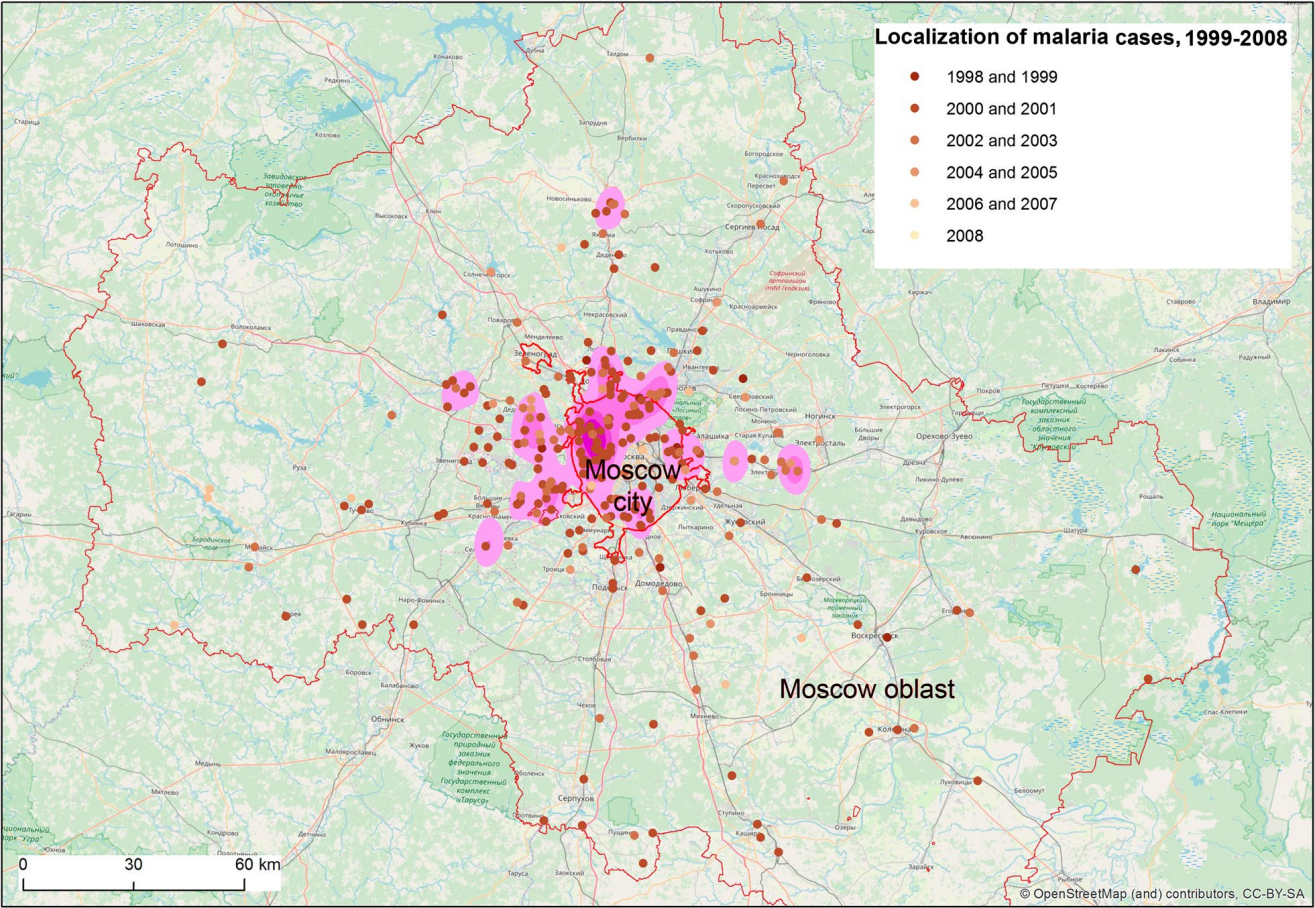
Spatial heterogeneity of malaria cases distribution in Moscow region, 1999–2008. Purple marks the areas with high compactness of autochthonous cases calculated by ArcGis kernel function

Between 1999 and 2008, 405 cases of vivax malaria transmission were recorded in Moscow region, of which 93 were observed within the borders of the city of Moscow (Fig. 2). Reported autochthonous cases of vivax malaria in Moscow region were unevenly distributed (Fig. 3). Within the boundaries of the city of Moscow, the bulk of the cases were concentrated in a continuous peripheral area in the western, northwestern and northern parts. It is worth noting that cases of malaria transmission were observed in the same locations during the previous post-eradication outbreaks in 1972 and 1981 [65]. This time, the greatest number of cases was registered not only in the aforesaid parts of Moscow city but in its immediate surroundings. Further to the east, in the northern part of Moscow city, the largest number of cases were confined to the valley of Yauza River, the main tributary of the Moskva River, and related ponds. In the east of the city, a few cases were confined almost exclusively to park and forest park areas. Finally, in the southern part of Moscow, all cases are mainly related to the Borisov ponds located on the Gorodnya River. In the central, most urbanized, part of Moscow and in the southeast part of the city where large industrial enterprises were located at the time of the outbreak, there were no recorded cases of malaria apart from a few large parks.

The area of the greatest concentration of malaria cases in the western part of the city of Moscow is an extension of the area of concertation of autochthonous cases outside the city (Krasnogorsk, Odintsovo and Istra districts). In that area, malaria transmission was recorded every year during the outbreak.

To the north of Moscow, the incidence was associated with the nearest satellite cities of Moscow (Mytishchi, Khimki, Dolgoprudny). The number of cases in smaller towns decreased in parallel with increasing distance from Moscow. The only relatively remote site (about 75 km to the north of Moscow) with a significant number (15) of cases was the town of Dmitrov with a few adjacent villages.

There were fewer autochthonous cases in the southern part of the Moscow region and they were also recorded mainly in the areas nearest to Moscow. The smallest number of cases was observed in the eastern part of the region.

### Dynamics of climate favourability for malaria transmission

The accumulated temperatures of 105 degree-days make it possible the maturation of one generation of *P. vivax* sporozoites, in other words, emergence of introduced cases cannot be excluded. When the sum surpasses 210 degree days, this is above the requirements for two consecutive generations of sporozoites, which is needed for emergence of indigenous cases, in addition to the introduced ones, that means the possibility of perpetuation of the transmission in the next year.

**Fig. 4:**
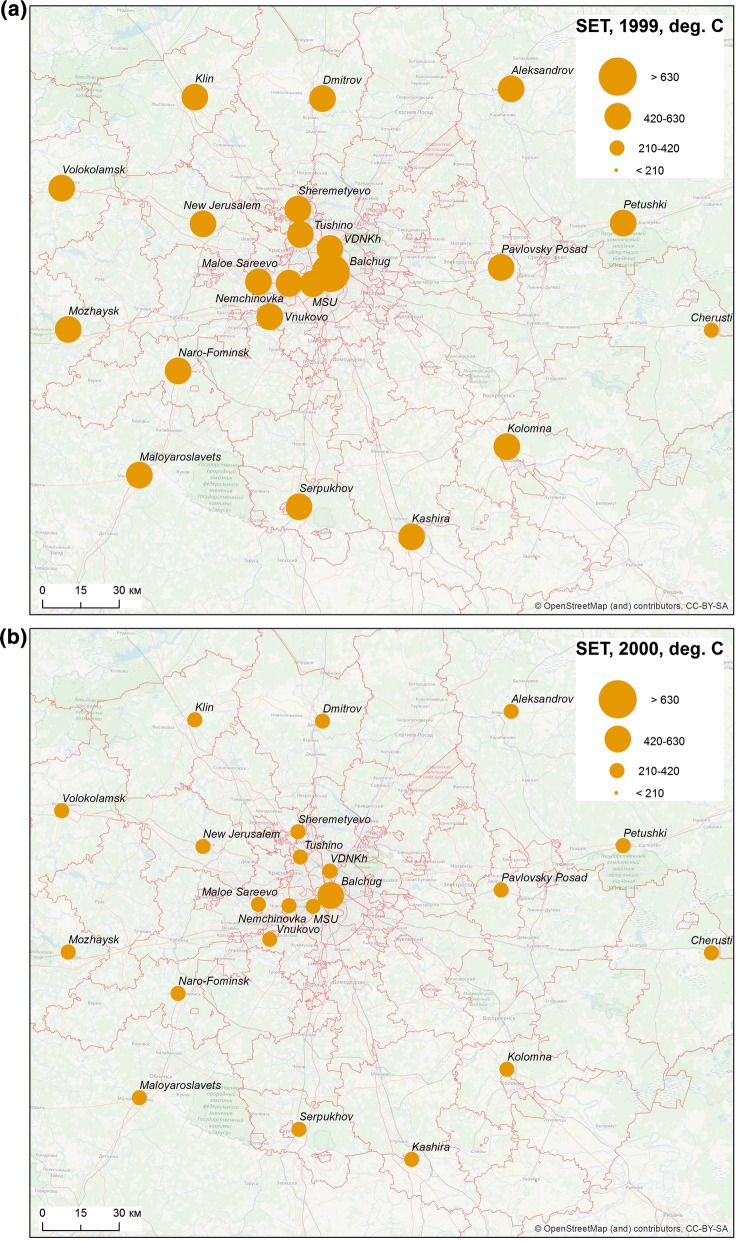

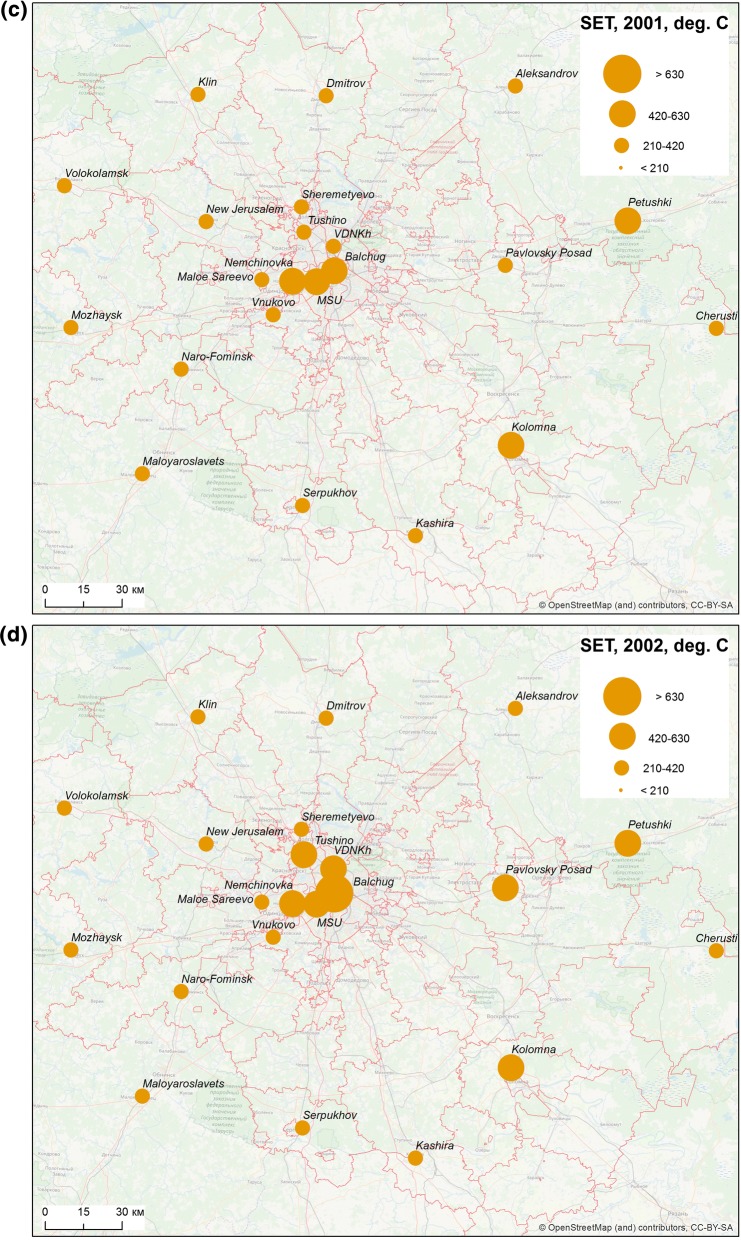

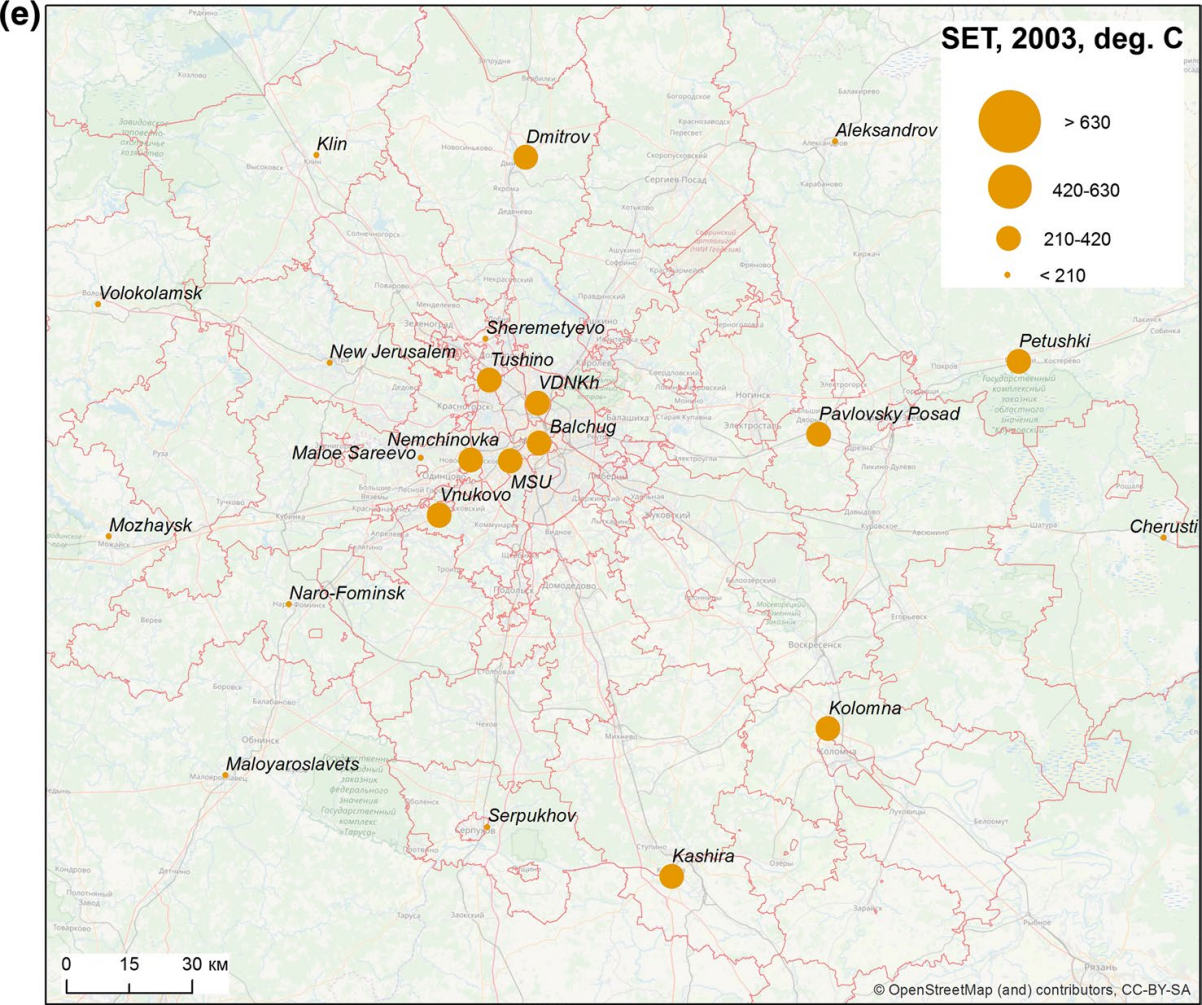
The sums of effective temperatures accumulated per season in the Moscow region, 1999–2003

The season of 1999 was characterized by a very high sum of effective temperatures unusual for Moscow region. On average, the sum of effective temperatures amounted to 444.8 degree-days in the Oblast. It exceeded 500 degree-days in some areas in the south of the region and surpassed the level of 700 degree-days in the centre of Moscow, due to UHI. After a slight decline in 2000, two hot summers followed, during which the sum of effective temperatures averaged 370 and 257 degree-days over the Oblast, in 2001 and 2002, respectively. For the city centre, this indicator reached 633 degree-days in 2002 (Fig. 4). Despite the significant urban–rural temperature differences, everywhere the sum of effective temperatures was significantly higher than 210 degree-days in 1999, 2001 and 2002. There was a significant decline to 200.6 degree-days in rural areas and 355.0 degree-days in the city in 2003, but in 2004 the average sums of temperatures rose again, up to 274.2 and 426.6 degree-days, respectively. In short, meteorological conditions in the city were favourable for a stable malaria transmission, whereas in rural areas, only the year of 2003 was unfavourable.

Spatial heterogeneity is noteworthy not only in sums of effective temperatures in Moscow megacity *versus* the surrounding suburbs and rural areas, but also in longer seasons of effective infectivity in the city (Table 2). However, during warm summers at the beginning and the peak of the outbreak (1999–2002), the season of effective infectivity was longer than 1 month both in rural and urban areas.

**Table 2:** The duration of the effective infectivity season in various localities of Moscow region and the surrounding areas (days)

Meteostation	Distance from Moscow centre, km, and the direction	1999	2000	2001	2002	2003	2004
Rural stations							
Dmitrov	66 N	50	51	37	42	20	25
Alexandrov	99 NE	44	52	36	37	16	18
Volokolamsk	109 NW	46	48	36	42	15	37
Mozhaysk	103 W	42	54	40	59	17	37
New Jerusalem	51 NW	44	49	38	40	15	41
Cherusti	152 E	54	54	38	35	20	43
Naro-Fominsk	68 SW	42	50	38	42	16	38
Serpukhov	94 S	42	57	43	61	17	44
Kolomna	103 SE	61	57	44	61	23	47
Klin	85 NW	50	53	36	37	13	22
Pavlovsky Posad	65 E	58	59	39	60	19	45
Petushki	117 E	58	59	41	50	24	47
Maloyaroslavets	110 SW	43	54	39	42	15	39
Kashira	107 s	55	56	42	44	20	–^a^
Nemchinovka	16 W	60	58	42	64	24	45
Sheremetyevo	30 NW	44	49	38	40	16	23
Vnukovo	30 SW	55	51	39	44	20	41
Small Sareevo	27 W	56	52	39	41	17	37
Urban stations (City of Moscow)							
Balchug (city center)	0	73	70	51	78	30	57
VDNHk (urban park)	9 N	62	59	41	62	21	41
MSU (urban park)	7 SW	63	61	42	74	24	48
Tushino	18 NW	59	55	39	61	20	41

^a^No data available

### Model of spatial distribution of malaria

Geospatial analysis of the relationship between cases of malaria transmission and each variables separately revealed very weak connection; there is no association with the absolute height of the area (r = − 0.004), it is weakly expressed with the density of cottage construction (r = 0.297) and is moderately expressed with the density of buildings (r = 0.465).

**Fig. 5:**
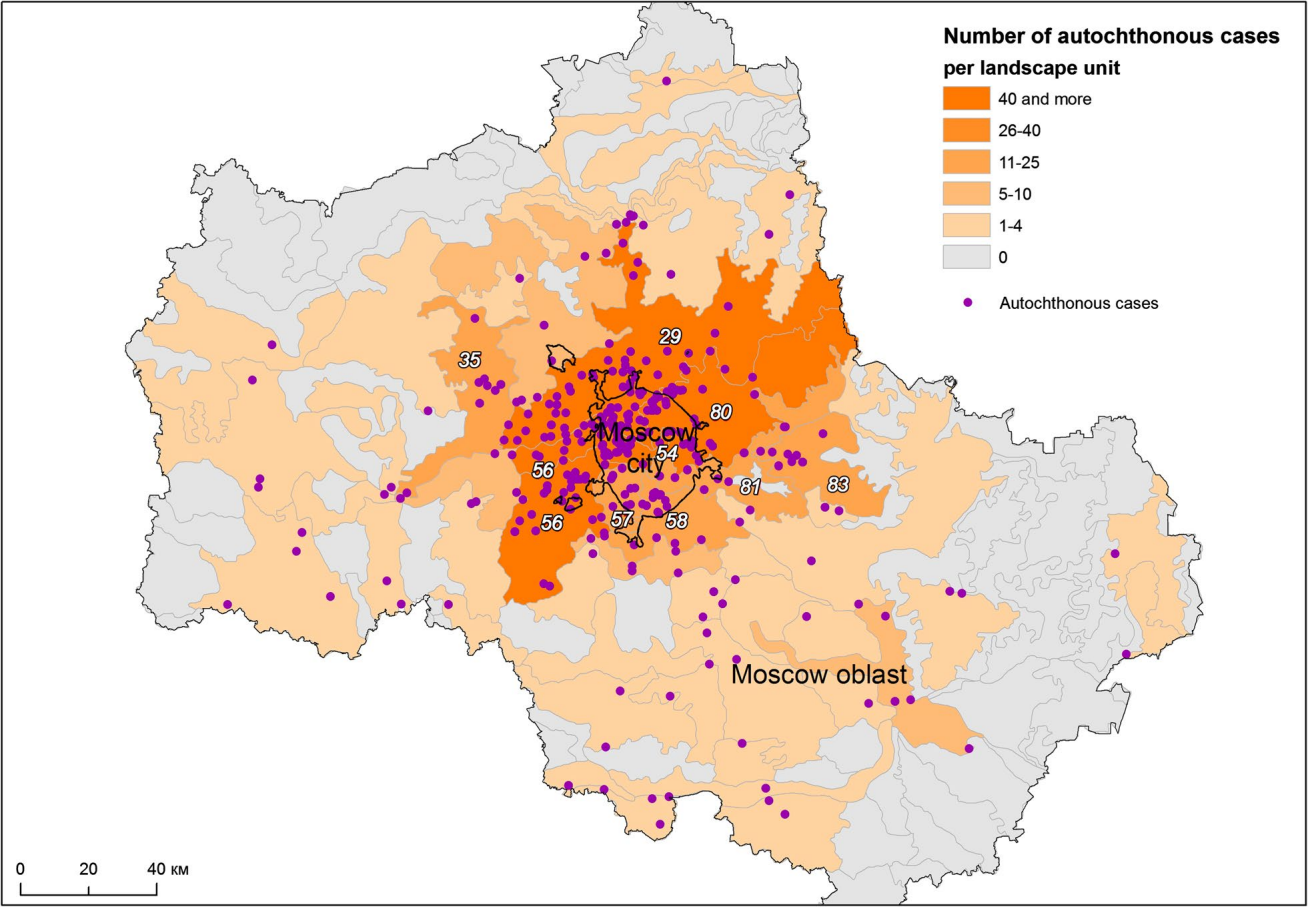
Distribution of malaria cases by landscape units The most affected landscape units are listed in Table 3

Most autochthonous cases of malaria are linked to four landscapes (No. 29—Moskvoretsko-Klyazminsky, No. 54—Moskvoretsky, No. 56—Aprelevsky-Kuntsevsky and No. 80—Shchelkovsky) (Table 3, Fig. 5). The names of the landscapes used in this work were borrowed from the inventory of landscape units [41]. The units were named by scientists in the course of the stratification to reflect the names of various prominent physiographic or urban objects.

**Table 3:** Relation of cases of malaria transmission to certain landscapes units

Landscape index [40]	Landscape name [40]	Number of cases of local transmission	Humidity pattern	Height above sea level, m
29	Moskvoretsko-Klyazminsky	77	Normal to very high	160–200
54	Moskvoretsky	46	Normal to very high	130–160
56	Aprelevsk-Kuntsevsky	45	Normal to very high	160–190
80	Schelkovsky	45	High and very high	140–150
81	Biserovsky	20	Increased to excess	130–140
83	Eleltrougolsky	17	Increased	140–150
35	Istrinsnsky	15	High and very high	170–200
58	Moskvoretsko-Bitsevsky	15	Normal	160–180
57	Teplostansky	13	Normal	180–200

A somewhat smaller number of cases belong to five more landscape areas directly adjacent to those three mentioned above. The remaining foci are usually represented by isolated malaria cases and are scattered over other landscape areas, and their number does not exceed 2 or 3 in each of them.

From the physiographical point of view, the landscape units that are good for malaria restoration belong to variants of moraine and water–ice plains with the humidity regime ranging from normal to excessive, with a predominance of elevated terrains at absolute altitudes from 120 to 200 m above sea level. Forest vegetation is largely replaced by arable land and garden plots as well as urban development areas. Among the residual vegetation, coniferous-deciduous forests prevail, as well as floodplains, upland meadows and occasional swamps. A distinctive feature of all landscapes is a strong degree of anthropogenic disturbance [66].

**Fig. 6:**
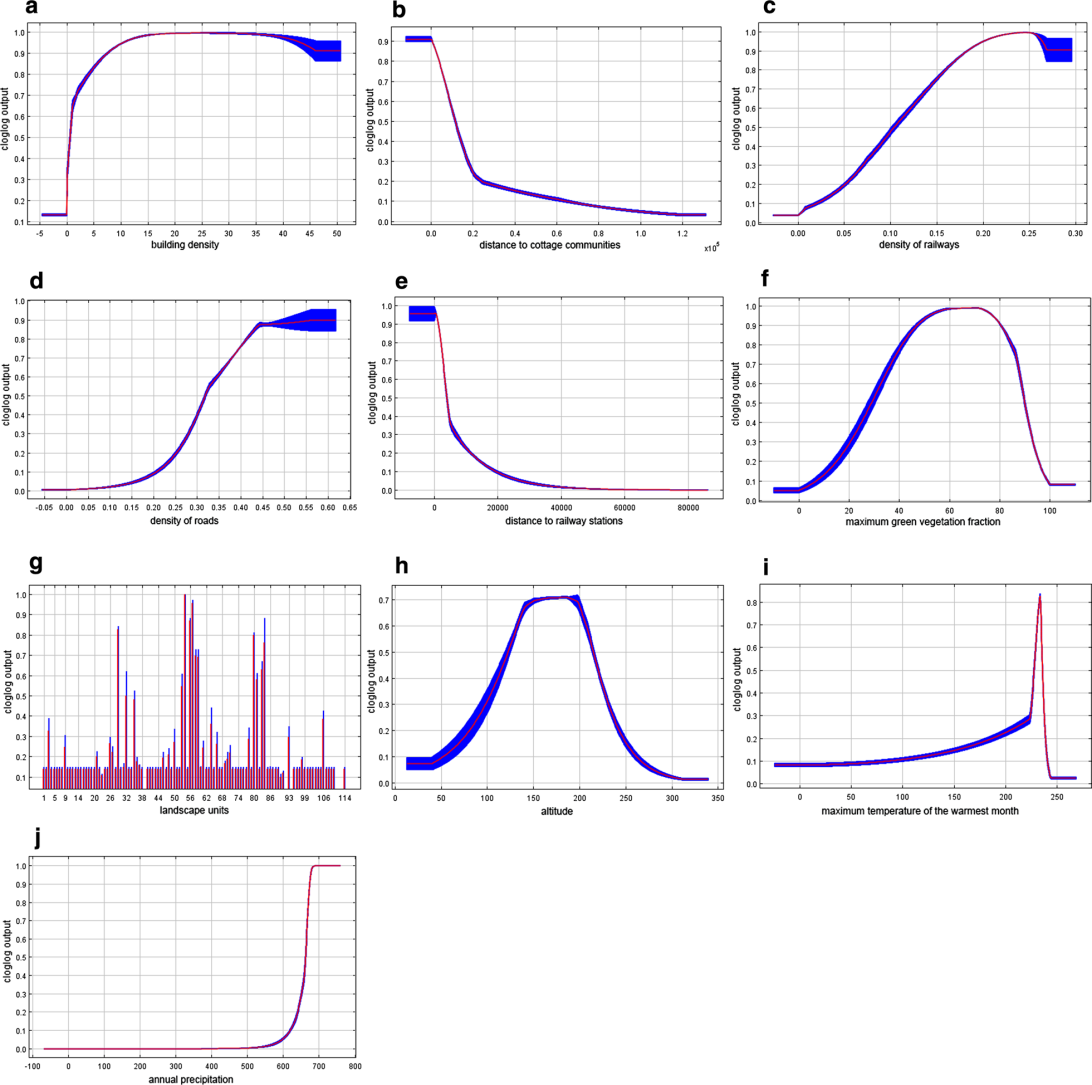
Response curves based on MaxEnt simulation results reflecting the influence of each of the significant spatial factors on the likelihood of appearance of autochthonous cases **a** building density; **b** distance to cottage communities; **c** density of railways; **d** density of roads; **e** distance to railway stations; **f** maximum green vegetation fraction; **g** landscape units; **h** altitude; **i** maximum temperature of the warmest month; **j** annual precipitation. Colour indicates mean value (red), standard deviation limits (blue)

More meaningful results were obtained when MaxEnt modelling was applied for multivariate analysis. The final MaxEnt model was calibrated with a regularization coefficient of 1.5. It demonstrates the AUC of 0.870 ± 0.015, which indicates a good predictive ability. Figure 6a–j shows the response curves for each variable demonstrating how the magnitude of the probability predicted by the model changes with the participation of only one given variable in the model.

**Fig. 7:**
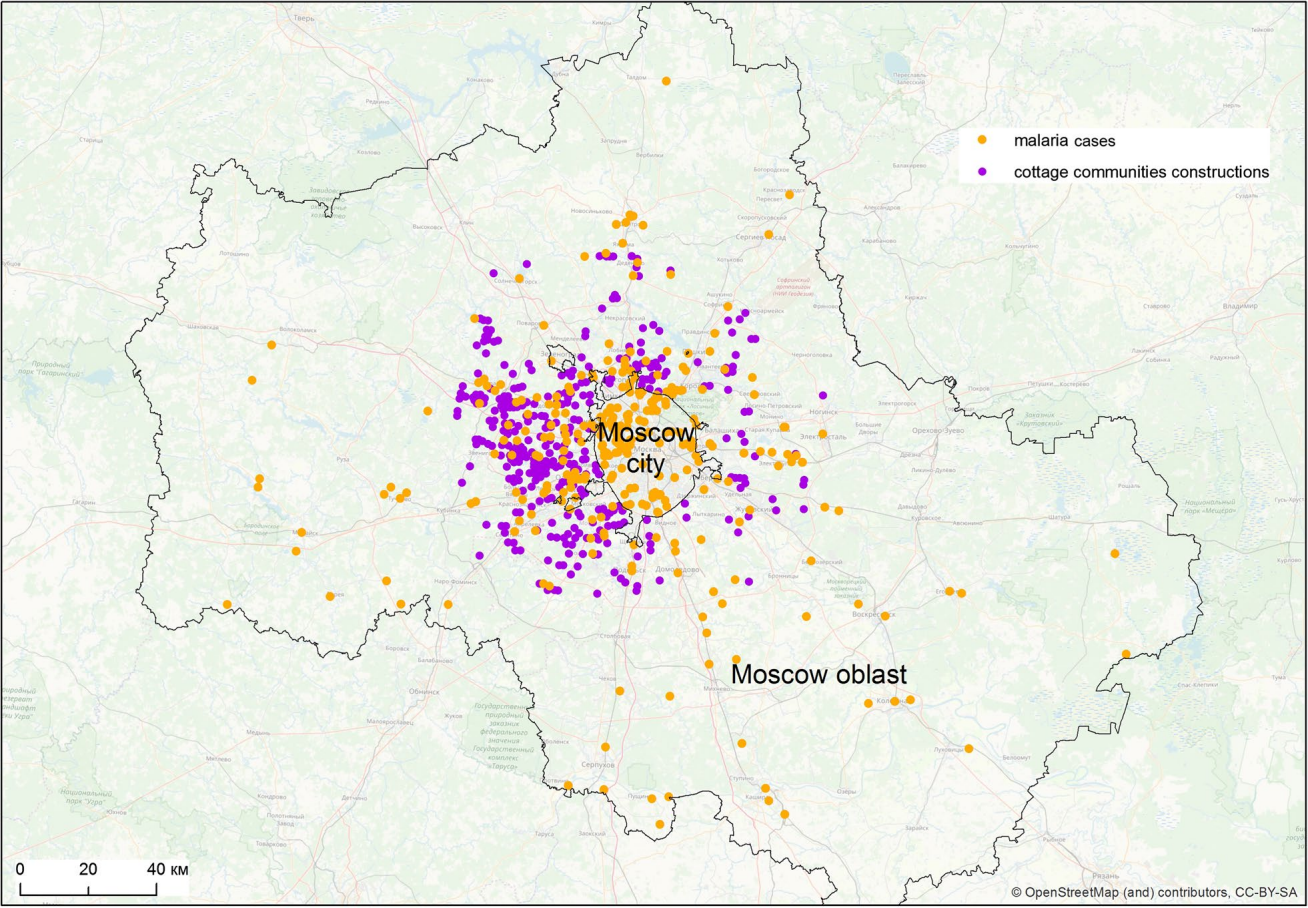
Distribution of malaria cases and cottage communities

The most significant factor in this modelling was building density that reflects populated areas, significance of 40.5% (Fig. 6a). The next most significant factor is the distance to the cottage communities: 25.1% (Fig. 6b). It demonstrates the expected relationship with the likelihood of outbreaks: it reaches maximum in a radius of about 2 km around the communities, significantly decreasing with increasing distance (Fig. 7).

The maximum likelihood of malaria cases is also positively associated with natural environmental variables: maximum green vegetation fraction (10.4%) and landscape units (9.1%) (Fig. 6f, g). For the maximum green vegetation fraction, a high probability response to values ranging from 50 to 80% is observed with a subsequent decrease in the response, which can be interpreted as the absence of malaria cases in places with too thin vegetation as well as too dense one. The highest response is demonstrated by landscapes with the index 57, 58, 56, 80, 29, 54, which generally corresponds to the distribution of the number of malaria cases observed over the landscape. The influence of altitude (1.6%) is of the least importance among environmental variables, with the highest response being in the range of height from 100 to 200 m (Fig. 6h).

The influence of mobility of the population on malaria cases is rather small (Fig. 6c–e): 6.9% for density of railways, less than 2% for density of roads and distance to railway stations. Nevertheless, the probability of outbreaks concentrated near railway stations decreases with the distance from them. Notably, malaria cases are not associated with population density. So, autochthonous cases occurred both in the cities of the Moscow region, where the population density is high, and in less densely populated rural areas.

The influence of climatic factors (maximum temperature of the warmest month and annual precipitation) on spatial heterogeneity of cases is less pronounced in the model (significance of 0.6 and 3.1%, respectively). Nevertheless, the dependence of the likelihood of an outbreak on the values of these variables is quite predictably increasing, which demonstrated an insignificant probability in the zone of low temperatures/precipitation and a sharply ascending probability with the increase in temperatures/precipitation. Noteworthy is the absence of the influence of low values of the variable in precipitation, which can be interpreted as the predominant attraction of malaria sites to wet locations (Fig. 6i, j). The possible impact of the local temperature features on the outbreak development is further discussed below.

**Fig. 8:**
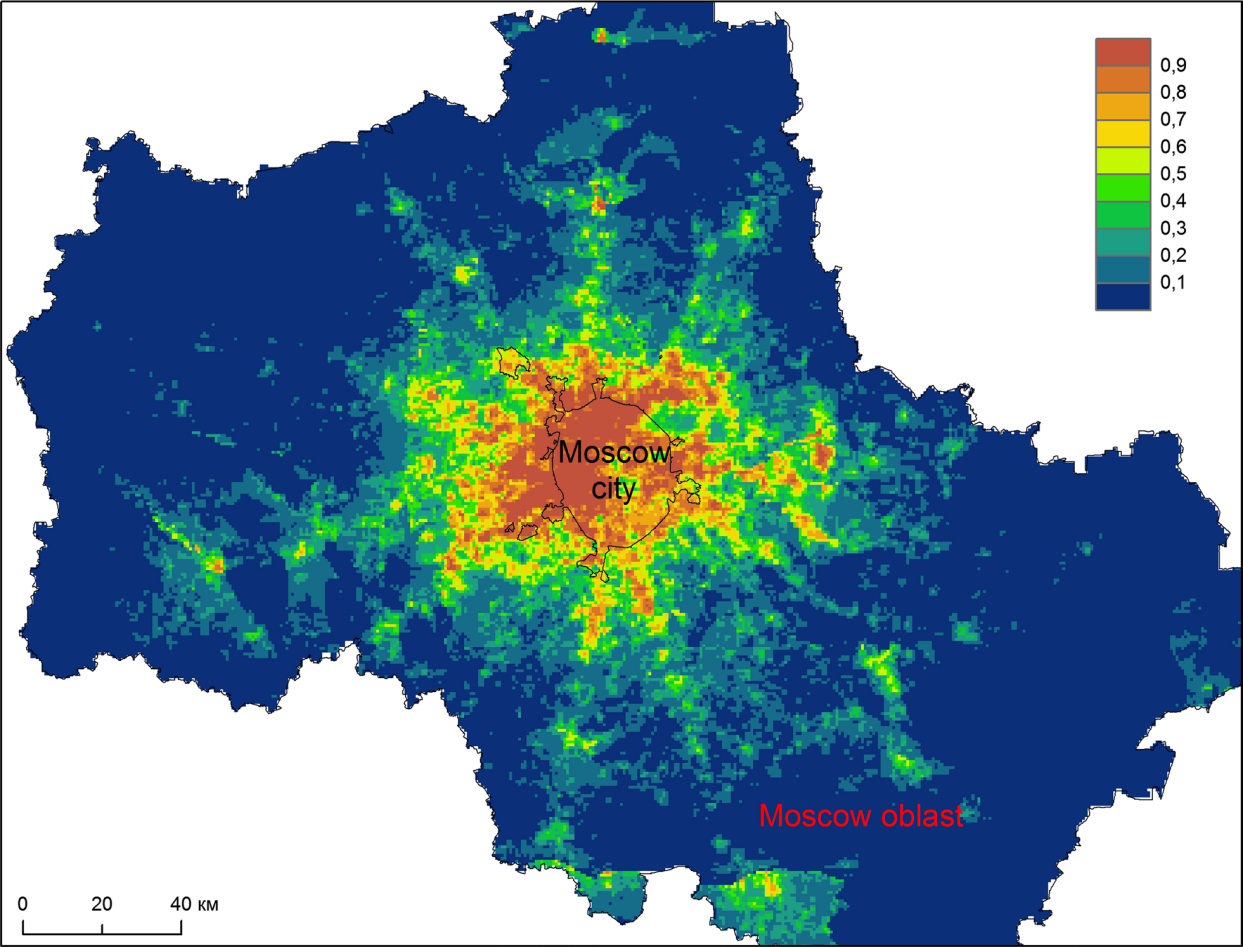
Modelling the degree of favourable conditions for the occurrence of malaria cases. Average values from 10 replications (red denotes a high degree of suitability; blue is a low degree). The values represent a probability that a set of explanatory variables within the certain cell is treated by the model as suitable for the emergence of a malaria case

Based on the modelling results, a map was created reflecting the territorial differentiation of Moscow region according to the suitability of infection re-emergence (Fig. 8). This map shows the statistically valid relationships between the distribution of autochthonous malaria cases and environmental and climatic factors. Malaria re-introduction is most likely to occur in rural area in the immediate vicinity of Moscow, along the main transport routes, and in satellite cities.

## Discussion

Malaria re-emerging after many years of the absence of its transmission is a subject of great interest to researchers and health authorities. Attempts to assess the environment in terms of predicting malaria re-introduction have been made regularly, especially for Europe where malaria has been eliminated [67], but has proven able to restore its transmission.

The most common approach to the assessment of possible malaria re-introduction is the analysis of distribution of vectors and changes in their bionomics [68, 69]. Climatic and social factors are often added [70, 71], but they are first considered from the point of view of their possible effect on vectors. However, in recent post-eradication outbreaks in Europe, Russia included, changes in vector factors have never been the trigger for the resumption of transmission, and re-appearance of autochthonous malaria was due to either intensification of its importation, or meteorological factors favouring the parasite maturation, or a combination thereof [72].

The approach implemented in this study is distinctive, as there is an attempt to contemplate the integrity of factors that may be involved. The results based on MaxEnt modelling showed that the combined influence of both natural and man-made environmental conditions played a key role in the Moscow outbreak.

Conceptually, the ability of parasitic diseases, including malaria, to thrive in a particular area (a malariogenic potential in case of malaria) may be expressed as a product of two parameters: receptivity and vulnerability [73]. Receptivity is the ability of ecosystems to incorporate the malaria parasite as their member [74, 75]. Vulnerability, or importation risk, is a measure of probability of the pathogen being imported from endemic areas, which is determined by the frequency, numbers and seasonality of gametocyte carrier arrival (less commonly, of infected mosquitoes). It is noteworthy that these terms have been recently reviewed by the newly formed Drafting Committee on Malaria Terminology. It was observed that the term of “vulnerability” may have several conflicting meanings in different medical sciences. Therefore, it has been suggested to replace it, as concerns the malaria terminology, by the “importation risk” [75]. This term is used throughout the text as a synonym of “vulnerability”.

In this study, the significance of the importation risk is corroborated by the observed high value (25.1%) of the parameter of the model called” distance to cottage communities”. This correlations in space can possibly be explained by the presence of labour migrants. During the studied period, the cottage communities were the sites of the most intensive construction activities in rural areas attracting huge numbers of labour migrants, mostly from Tajikistan [16, 76], which was the scene of major post-eradication outbreaks at that time [77]. Arrival of seasonal workers and illegal migrants in Russia from some countries of the CIS adversely affected the malaria situation. There were no reliable figures on the actual size of illegal immigration in Russia, as estimations differed significantly from official registration data. Some researchers estimated the number of illegal immigrants in Russian Federation in late nineties between 400,000 and 7 million, and in Moscow alone there were no less than one million from the “near abroad” [78]. Migration control was very weak in late nineties in Russia, which contributed to an increase of imported cases from 218 in 1990 to 1042 in 1998 [76].

Migrants coming from the areas of intensive transmission were often more knowledgeable about malaria than Moscow medics did. Some of them had had malaria in the recent past and had residual immunity that mitigated malaria in case of new infections. Many had antimalarials in their possession. They knew that in case of positive diagnosis they would be subjected to a long hospitalization, which was not in their interest. Illegal or semi-legal existence of many of them and absence of medical insurance prevented them from seeking medical care. At the same time, local medical facilities were not interested in delivering services to migrants [16]. As the result, detection of malaria among migrants was very incomplete.

Migrants were aggregated mostly in construction of housing in cities and summer houses for urban dwellers in the countryside. During the construction work in the countryside, usually in summers, they dwelled, as a rule, in semi-constructed structures open to attacks from vectors.

The association of autochthonous malaria and cottage construction seems to corroborate the hypothesis that migrants were the major source of malaria. A similar situation was observed during some of the other post-eradication outbreaks in Europe, where labour migrants and refugees were at the origin of renewed malaria transmission, for example, in Bulgaria [22] and Greece [12].

The receptivity of ecosystems is determined by several natural factors. It is generally recognized that the main factor in temperate areas are summer temperatures (while rainfall may be of decisive importance in sub-tropics and tropics) [79]. An outbreak of vivax malaria in Moscow region has developed amid exceptionally favourable weather conditions for the vivax pathogen’s extrinsic development. It was previously demonstrated that by the start of the 21st Century, conditions for malaria transmission in Moscow region improved compared with 1970–80s [51]. The most dramatic changes in the sums of effective temperatures and duration of season of effective mosquito infectivity have occurred since the mid-1990s, which is consistent with general climatic warming in Moscow region [27]. The highly favourable conditions existing during two consecutive seasons (2001 and 2002) might have a crucial significance for the formation of a steady infection reservoir, due to an unusual accumulation of hypnozoite carriers.

The role of the climatic factor in malaria transmission in the model is not so clearly traced. It may be caused firstly by temperature conditions that were uniformly favourable for the development of *P. vivax* throughout the region (urban and rural areas) during the beginning of the outbreak. Secondly, there is a relatively small variation in the considered variables of maximum temperature of the warmest month according to the gridded WorldClim data. The local climate features, such as UHI, are weakly pronounced by maximum temperatures that are observed at daytime [26]. More information can give daily mean and minimum temperature that are more sensitive to local climate features. Unfortunately, available datasets do not allow explicit consideration of the urban anomaly of air temperature and temperature-dependent malaria indicators in the MaxEnt model. The network of weather stations in Moscow region is too sparse for such a task, while globally available gridded temperature datasets are unable to adequately represent temperature heterogeneity in urban areas [46]. Development of detailed and reliable climatic datasets is essential for better understanding of epidemiological threats in urban areas.

More detailed analysis of the sums of effective temperatures and the duration of season of effective infectivity based on temperature data from rural and urban weather stations, shows a spatial heterogeneity that is clearly noted in the distribution of these indicators. This is a clear manifestation of the UHI effect. The influence of UHI is expressed in significantly higher values of indicators in the city centre compared to city parks and the countryside. The year 2003 illustrates that heterogeneity of the territory is manifested mainly during unfavourable weather conditions. That year the sums of effective temperatures, needed for the development of the parasite could be accumulated only in Moscow city (due to the effect of UHI) and in the east of the Oblast. As a result, the number of cases was considerably less in 2003 than in very favourable 2001 and, especially, 2002. In warm years, when the thermal conditions are rather uniform across the region, there is not much difference between the areas under the influence of UHI and the periphery, whereas this difference provokes a sharp decrease of cases at cooler areas during malaria’s lean years.

It can be assumed that in conjunction with human-related environmental factors, the urban–rural temperature difference may explain why areas most affected by malaria were located at the peripheral districts of the city (northwest of Moscow) or near city borders. On the one hand, these are the territories that are most attractive in terms of building density, reflecting areas of population concentration, and more suitable places for anopheline breeding in comparison with the city centre while, on the other hand, the temperatures in these built-up areas are more favourable for the development of the pathogen in comparison with rural areas. In addition, it was there that most large-scale cottage construction unfolded. Despite that, UHI was not represented explicitly in the MaxEnt model, its influence should not be excluded.

The influence of landscape on the distribution of endemic malaria is, in general, well known [79–81]. The issue of the influence of landscape division on malaria re-introduction in temperate latitudes is not so well studied because there are not so many well-documented cases of such re-emergence. An assessment of environmental suitability for malaria transmission in Greece [82], which used several climatic and environmental parameters, showed that the highest risk of malaria transmission was confined to particular landscapes of coastal recreational zones.

In Moscow region, some landscapes are especially prone to re-introduction of malaria. The role of the landscape factor is demonstrated by an accumulation of cases in and around the city of Dmitrov, 75 km north of Moscow, an area of a remote protuberance of the most malaria-prone landscape, Moskvoretsko-Klyazminsky (No 29), that lies amid the least affected landscapes. It is noteworthy that the territories inside the city of Moscow and beyond belonging to the same landscape unit have similar malariogenic potential, despite the effects of the urbanization.

Suitability of particular types of landscape for malaria transmission may change over time. For example, the areas in the east of the region which were most affected at the beginning of the 20th Century [9, 10], were not affected during the outbreak a century later. In this case, the cessation of peat mining and the subsequent land reclamation in the 1950s played a decisive role. In addition, in the eastern part of the Moscow agglomeration, intensive industrial development took place during the Soviet period which produced considerable industrial pollution creating an environment that was unfavourable for malaria vectors. Similarly, in industrial zones within Moscow city, there were very few cases of malaria transmission during the recent malaria re-emergence.

In Moscow region, malaria re-introduction begins firstly in the landscape units with elevated humidity in well-drained territories, gravitating to the valleys of relatively large rivers. This agrees with a long-standing observation by Favr that malaria is a disease of river valleys in Central Russia [7]. At the turn of the 19th Century, this assumption was widespread among Muscovites, as evidenced by A.P.Chekhov, the famous writer, who was also a medical practitioner in Melikhovo, 75 km south of Moscow. He notes in a letter to A. S. Suvorin on 1 April, 1897 that “Melikhovo is a healthy place; it is just on the watershed, it stands high, so there is never a fever in it.” [83].

The main water artery in the area is the Moskva River which crosses the megacity from northwest to southeast. Its waters are relatively clean on their entry in the city but become polluted by industrial and municipal discharges downstream. Because of this gradient of pollution, the rural districts bordering Moscow city from the northwest and west (that were most affected by malaria in 1999–2008) are more suitable for anopheline breeding. At the same time, those areas have greater recreational attractiveness, and were the scene of extensive construction of cottage communities at the turn of the century.

Further to the periphery, in relatively remote areas that do not attract numbers of migrants, malaria transmission is mostly associated with moist river valleys. However, in some of those areas conditions for malaria transmission worsened due to land reclamation/drainage and industrial and possibly domestic pollution.

The altitude of the terrain is a well-known factor of malaria [79, 84–86]. Its importance has been demonstrated recently in Greece with respect to the suitability of territories for malaria transmission [82] and in Iran with respect to the distribution of the most important vectors species [60]. Despite the fact that the response of probability of the model did not show high significance (it may happen due to more pronounced influence of other variables in the model, such as distance to cottage communities, indirectly reflecting the spread and concentration of labour migrants), the curve of the distribution of cases by altitude (Fig. 6h) shows that the cases were confined to a range of 100–200 m. In addition, the most affected landscapes (Table 3) are linked to the same altitudes. It may be assumed that such model response is due to insufficient number of malaria transmission cases and a small range of absolute heights. In Moscow region, this factor has no particular meaning below 200 m, a cut-off level, above which the re-establishment of malaria is least probable.

Although the receptivity of the Moscow region may be deemed medium [73], the risk of importation of *P. vivax* (a species most adapted to the local vectors) is very high, since Moscow is becoming attractive to economic migrants [77]. As a result, malariogenic potential, which is the product of receptivity and the risk of importation is quite high, irrespective to the latitude.

Whereas the historic post-elimination outbreaks (including those in Russia in the 1970s and 1980s) involved limited territories, this particular outbreak in Central Russia is unique in terms of its great extent and variety of ecosystems involved.

The loss of interest for malaria control was one more reason for the recent malaria re-introduction in Moscow region. After the last autochthonous cases in the 1980s related to the Afghan war [87] malaria re-introduction ceased to be regarded as a significant threat to public health which led to inadequate staffing, especially of medical entomologists. Despite the high malariogenic potential, the epidemic waned as soon as importation of new cases from ex-Soviet republics came to the stop around 2009 [77]. Even though the summer of 2010 was extremely hot [88], no transmission of malaria occurred at that time.

## Conclusions

One of the contributing factors for the re-introduction of malaria in Moscow region in 1999–2008 was highly intensive importation, coupled with a decrease in epidemiological awareness against the background of extremely favourable meteorological conditions during 1999, 2001, and 2002. However, both in the past and at the present time, a combination of natural and human-related factors, as well as territorial heterogeneity in relation to malaria transmission affects the development of outbreaks.

In the conditions prevailing in the opening years of the 21st Century, the malariogenic potential in relation to vivax malaria was high in Moscow region, albeit heterogeneous in this regard. During the 1999–2008 outbreak the most affected areas were those with a high concentration of population.

A significant role was also played by rural construction (the attraction of labour migrants from Tajikistan, among whom there were many parasite carriers, including asymptomatic ones), vegetation density, belonging to a particular landscape division, and the altitude below 200 m above sea level. Most likely, the intensive UHI of Moscow megacity was an additional factor which amplified the outbreak in urban and suburban areas. In the future, in case of a renewed massive importation, it is in Moscow that the most favourable conditions are expected to be existing for vivax malaria re-introduction, compared with other regions of Russia.

## Data Availability

All documents and publications in Russian are available at the Lomonosov Moscow State University, Moscow, Russian Federation.

## Abbreviations

ADT: Average daily temperature
ASCII: American standard code for information interchange
AUC: Area under the curve
CIS: Commonwealth of independent states
MSU: Moscow State University
RIHMI-WDC: Russian institute for hydrometeorological information—world data center
ROC: Receiver operating characteristic
UHI: Urban heat island
USSR: Union of the soviet socialist republics
VDNKh: *Vystavka Dostizheniy Narodnogo Khozyaystva*, exhibition of achievements of national economy
WHO: World Health Organization

## References

[1] Beljaev AE, Rybalka VM, Lysenko AJ, Abrashkin-Zhuchkov RG, Alekseeva MI, Arsenyeva LP, et al. *Plasmodium vivax*: further observations on polymorphism in relation to the duration of exo-erythrocytic development (In Russian). In Malaria Parasites of Mammals; Academy of sciences of the USSR Protozoology series; Leningrad: Nauka; 1986: 11; 40–157. **(In Russian)**.

[2] Reiter P (2000). From Shakespeare to Defoe: malaria in England in the little ice age. Emerg Infect Dis..

[3] Lindsay S, Joyce A (2000). Climate change and the disappearance of malaria from England. Global Change Hum Health..

[4] Bruce-Chwatt LJ, de Zulueta J (1980). The rise and fall of malaria in Europe. A historico-epidemiological study.

[5] Hulden L, Hulden L (2009). The decline of malaria in Finland–the impact of the vector and social variables. Malar J..

[6] Vasiliev KG, Segal AE (1960). The history of epidemics in Russia.

[7] Favr VV. Study of malaria in Russia from the public health angle. Kharkov: 1903. **(in Russian)**.

[8] Sergiev PG, Dukhanina NN, Demina NN, Shipitsina NK, Ozeretskovskaya NN, Lysenko AY. Malaria. A multi-volume guide to microbiology, clinic and epidemiology of infectious diseases. Moscow: Medicine; 1968: 9; 37–115. **(in Russian)**.

[9] Skibnevsky AI. Materials on the incidence of specific diseases vol. 2: The spread and manifestation of malaria in the population of the Moscow governorate. Moscow: 1903. **(in Russian)**.

[10] Oganov LI. Malaria in the Moscow region and its seasonal periodicity. PhD Thesis, 1947. Martsinovski Institute for Medical Parasitology and Tropical Medicine, Moscow, USSR. **(in Russian)**.

[11] WHO. Malaria in the WHO European region. Fact sheet. 2016. http://www.euro.who.int/__data/assets/pdf_file/0009/246168/Fact-sheet-Malaria-Eng.pdf?ua=1. Accessed 08 Oct 2019.

[12] Danis K, Lenglet A, Tseroni M, Baka A, Tsiodras S, Bonovas S (2013). Malaria in Greece: historical and current reflections on a re-emerging vector borne disease. Travel Med Infect Dis..

[13] de Zulueta J, Ramsdale CD, Coluzzi M (1975). Receptivity to malaria in Europe. Bull World Health Organ.

[14] WHO. Regional strategy: from malaria control to elimination in the WHO European Region 2006–2015. Copenhagen: World Health Organization Regional Office for Europe, 2006. http://www.euro.who.int/__data/assets/pdf_file/0011/98750/E88840.pdf. Accessed 8 Oct 2019.

[15] Kondrashin AV, Morozova LF, Stepanova EV, Turbabina NA, Maksimova MS, Morozov EN (2018). On the epidemiology of *Plasmodium vivax* malaria: past and present with special reference to the former USSR. Malar J..

[16] Mironova VA, Beljaev AE, Yushchenko GV (2011). Migration processes and malaria in Russia. Current issues in the epidemiology of infectious diseases: Shaposhnikov AA.

[17] Ambroise-Thomas P, Quilici M, Ranque P (1972). Réapparition du paludisme en Corse. Bull Soc Pathol Exot.

[18] Armengaud A, Legros F, D’Ortenzio E, Quatresous I, Barre H, Houze S (2008). A case of autochthonous *Plasmodium vivax* malaria, Corsica, August 2006. Travel Med Infect Dis.

[19] Toty C, Barré H, Le Goff G, Larget-Thiéry I, Rahola N, Couret D (2010). Malaria risk in Corsica, former hot spot of malaria in France. Malar J..

[20] Baldari M, Tamburro A, Sabatinelli G, Romi R, Severini C, Cuccagna G (1998). Malaria in Maremma, Italy. Lancet..

[21] Santa-Olalla Peralta P, Vazquez-Torres MC, Latorre-Fandos E, Mairal-Claver P, Cortina-Solano P, Puy-Azon A (2010). First autochthonous malaria case due to *Plasmodium vivax* since eradication, Spain, October 2010. Euro Surveill.

[22] Kurdova R, Vutchev D, Petrov P (2001). Malaria situation in Bulgaria and surveillance measures (1991–2000). Global Nest Intern J..

[23] Kampen H, Maltezos E, Pagonaki M, Hunfeld K-P, Maier W, Seitz H (2002). Individual cases of autochthonous malaria in Evros Province, northern Greece: serological aspects. Parasitol Res.

[24] Olaso A, Ramos JM, López-Ballero MF, Olaso I (2017). Malaria in Europe: follow-up of autochthonous malaria in Greece and new risks. Enferm Infecc Microbiol Clin.

[25] http://www.gks.ru/wps/wcm/connect/rosstat_main/rosstat/ru/statistics/population/demography/ Accessed 11 Sep 2019.

[26] Oke TR, Mills G, Christen A, Voogt JA (2017). Urban climates.

[27] Kislov AV (2017). Climate of Moscow in the context of global warming.

[28] Lokoshchenko MA (2014). Urban “heat island” in Moscow. Urban Clim..

[29] Varentsov M, Wouters H, Platonov V, Konstantinov P (2018). Megacity-induced mesoclimatic effects in the lower atmosphere: a modeling study for multiple summers over Moscow, Russia. Atmosphere..

[30] Varentsov MI, Grishchenko MY, Wouters H. Simultaneous assessment of the summer urban heat island in Moscow megacity based on in situ observations, thermal satellite images and mesoscale modeling. Geography, Environment, Sustainability. 2019; https://ges.rgo.ru/jour/article/view/762. Accessed 26 Dec 2019. 10.24057/2071-9388-2019-10.

[31] Kislov AV, Varentsov MI, Gorlach IA, Alekseeva LI (2017). The heat island” of the Moscow agglomeration and the urban increase of global warming. Bull Mosc Univ Geogr..

[32] Gornostaeva RM, Danilov AV (1999). Mosquitoes of Moscow and the Moscow region.

[33] Artemiev MM, Baranova AM, Darchenkova NN, Dremova VP, Ganushkina LA, Markovich NY (2000). The malarial mosquitoes of Russia the genus Anopheles. Med Parazitol (Mosk)..

[34] Gordeev MI, Ejov MN, Zvantsov AB, Perevozkin VP (2005). Malaria mosquitoes in Moscow and in the Moscow Region: cytogenetic analysis. Med Parazitol (Mosk)..

[35] Moskaev AV, Gordeev MI, Kuzmin OV (2015). Chromosomic composition of populations of the mosquito *Anopheles messeae* in the center and periphery of its geographical range. Vestnik MGOU. Nat Sci..

[36] WHO (2010). Practical guidelines on malaria elimination in countries of WHO European region.

[37] WorldClim–Global climate data. http://worldclim.org/www.worldclim.org.

[38] Hijmans RJ, Cameron SE, Parra JL, Jones PG, Jarvis A (2005). Very high resolution interpolated climate surfaces for global land areas. Int J Climatol.

[39] The USGS land cover institute https://archive.usgs.gov/archive/sites/landcover.usgs.gov/green_veg.html). Accessed 26 Aug 2019.

[40] Broxton P, Zeng X, Scheftic W, Troch P (2014). A MODIS-based global 1-km maximum green vegetation fraction dataset. J Appl Meteorol Climatol..

[41] Mamai II (1997). Landscapes of the Moscow region and their current state.

[42] Makhrova AG (2008). Organized cottage communities: a new type of settlement (on the example of the Moscow region). Reg Stud..

[43] Macdonald G (1957). The epidemiology and control of malaria.

[44] Lysenko AJ, Semashko IN. Geography of malaria: a medical-geographical study of an ancient disease. Itogi nauki: meditsinskaya geografija. Moscow: VINITI, 1968;5-146. English translation. **(in Russian)**. https://www.who.int/malaria/publications/atoz/lysenko.pdf?ua=1. Accessed 20 Nov. 2019.

[45] Russell P, West L, Manwell R, Macdonald G (1963). Practical malariology.

[46] Morgan B, Guénard B (2019). New 30 m resolution Hong Kong climate, vegetation, and topography rasters indicate greater spatial variation than global grids within an urban mosaic. Earth Syst Sci Data..

[47] Beklemishev VN (1947). The problem of typization of malaria foci and some types of malariogenic landscapes. Med Parazitol (Mosk)..

[48] Moshkovsky SD (1946). The dependence upon temperature of the speed of development of malaria plasmodia in the mosquito. Med Parazitol (Mosk)..

[49] Bodenheimer FS (1925). On predicting the development cycles of insects. I. *Ceratitis capitata* Wied. Bull Soc R Entomol Egypt..

[50] WHO (2007). Guidelines on prevention of the reintroduction of Malaria.

[51] Mironova VA, Shartova NV, Beljaev AE, Varentsov MI, Grishchenko MY (2019). Effects of climate change and heterogeneity of local climates on the development of malaria parasite (*Plasmodium vivax*) in Moscow megacity region. Int J Environ Res Public Health..

[52] Phillips S, Anderson R, Schapire R (2006). Maximum entropy modeling of species geographic distributions. Ecological Model..

[53] Peterson AT, Bauer JT, Mills JN (2004). Ecologic and geographic distribution of filovirus disease. Emerg Infect Dis.

[54] Rose H, Wall R (2011). Modelling the impact of climate change on spatial patterns of disease risk: sheep blowfly strike by *Lucilia sericata* in Great Britain. Int J Parasitol.

[55] Du Z, Wang Z, Liu Y, Wang H, Xue F, Liu Y (2014). Ecological niche modeling for predicting the potential risk areas of severe fever with thrombocytopenia syndrome. Int J Infect Dis..

[56] Abdrakhmanov SK, Mukhanbetkaliyev YY, Korennoy FI, Sultanov AA, Kadyrov AS, Kushubaev DB (2017). Maximum entropy modeling risk of anthrax in the Republic of Kazakhstan. Prev Vet Med..

[57] Abdrakhmanov SK, Sultanov AA, Beisembayev KK, Korennoy FI, Kushubaev DB, Kadyrov AS (2016). Zoning the territory of the Republic of Kazakhstan as to the risk of rabies among various categories of animals. Geospat Health.

[58] Mwakapeje ER, Ndimuligo SA, Mosomtai G, Ayebare S, Nyakarahuka L, Nonga HE (2019). Ecological niche modeling as a tool for prediction of the potential geographic distribution of *Bacillus anthracis* spores in Tanzania. Int J Infect Dis..

[59] Escobar LE, Craft ME (2016). Advances and limitations of disease biogeography using ecological niche modeling. Front Microbiol..

[60] Hanafi-Bojd AA, Sedaghat MM, Vatandoost H, Azari-Hamidian S, Pakdad K (2018). Predicting environmentally suitable areas for *Anopheles superpictus* Grassi (s.l.), *Anopheles maculipennis* Meigen (s.l.) and *Anopheles sacharovi* Favre (Diptera: Culicidae) in Iran. Parasit Vectors..

[61] Brown JL, Bennett JR, French CM (2017). SDMtoolbox 2.0: the next generation Python-based GIS toolkit for landscape genetic, biogeographic and species distribution model analyses. PeerJ..

[62] Pearson RG. Species’ distribution modeling for conservation educators and practitioners. In: lessons in conservation, 2010; 3:54–89. https://www.amnh.org/research/center-for-biodiversity-conservation/resources-and-publications/lessons-in-conservation/lessons-in-conservation-volume-iii. Accessed 20 Dec 2019.

[63] Araújo MB, Pearson RG, Thuiller W, Erhard M (2005). Validation of species-climate impact models under climate change. Glob Change Biol.

[64] Merow C, Smith M, Silander J (2013). A practical guide to MaxEnt for modeling species’ distributions: what it does, and why inputs and settings matter. Ecography.

[65] Mironova VA. Geographical determinants of malaria re-introduction in different ecosystems: evaluation and prognosis. Ph.D. thesis. Moscow: Lomonosov Moscow State University; 2006. **(in Russian)**.

[66] Moscow Region: History, Culture, Economics. Moscow, IPC “Dizain. Informatsiya. Kartografiya”. 2004. **(in Russian)**.

[67] WHO. World malaria report 2018. Geneva: World Health Organization; 2018. https://www.who.int/malaria/publications/world-malaria-report-2018/en/. Accessed 26 Nov 2019.

[68] Kavran M, Zgomba M, Weitzel T, Petric D, Manz C, Becker N (2018). Distribution of *Anopheles daciae* and other *Anopheles maculipennis* complex species in Serbia. Parasitol Res.

[69] Tagliapietra V, Arnoldi D, Di Luca M, Toma L, Rizzoli A (2019). Investigation on potential malaria vectors (*Anopheles* spp.) in the province of Trento, Italy. Malar J..

[70] Linard C, Ponçon N, Fontenille D, Lambin EF (2009). A multi-agent simulation to assess the risk of malaria re-emergence in southern France. Ecol Model.

[71] Pergantas P, Tsatsaris A, Malesios C, Kriparakou G, Demiris N, Tselentis Y (2017). A spatial predictive model for malaria resurgence in central Greece integrating entomological, environmental and social data. PLoS ONE.

[72] Cohen JM, Smith DL, Cotter C, Ward A, Yamey G, Sabot OJ (2012). Malaria resurgence: a systematic review and assessment of its causes. Malar J..

[73] WHO. Regional office for Europe. Receptivity to malaria and other parasitic diseases: report on a WHO working group, Izmir, 11–15 September 1978. Copenhagen, WHO Regional Office for Europe, “1979”. https://apps.who.int/iris/handle/10665/204466/ Accessed 20 Oct 2019.

[74] Lysenko AY, Kondrashin AV, Ejov MN (2003). Malariology.

[75] WHO. WHO malaria terminology. 2016 (updated March 2018). https://www.who.int/malaria/publications/atoz/malaria-terminology/en/ Accessed 12 Nov 2019.

[76] Syskova TG. The impact of migration on the incidence of parasitic diseases and the development of preventive measures. Synopsis of a Ph.D. thesis, 2005. Martsinovski Institute for Medical Parasitology and Tropical Medicine, Moscow.

[77] Ejov MN, Sergiev VP, Baranova AM, Kurdova-Mintcheva R, Emiroglu N, Gasimov E (2017). Malaria in the WHO European region. On the road to elimination, 2000–2015.

[78] Krassinets E. Illegal migration and employment in Russia. Informal network on foreign labour in Central and Eastern Europe, ILO/Luxemburg co-operation: project rer/97/mo2/lux. https://www.ilo.org/wcmsp5/groups/public/—ed_protect/—protrav/—migrant/documents/publication/wcms_201973.pdf. Accessed 12 Feb 2020.

[79] Beljaev AE, Casman EA, Dowlatabadi H (2002). Determinants of malaria in the middle East and North Africa. The contextual determinants of malaria.

[80] Kitron U (1998). Landscape ecology and epidemiology of vector-borne diseases: tools for spatial analysis. J Med Entomol.

[81] Schapira A, Boutsika K (2012). Malaria ecotypes and stratification. Adv Parasitol.

[82] Sudre B, Rossi M, Van Bortel W, Danis K, Baka A, Vakalis N (2013). Mapping environmental suitability for malaria transmission, Greece. Emerg Infect Dis..

[83] Chekhov AP (1957). Collected Works. Moscow. Goslitizdat.

[84] The relation of malaria to altitude. Lancet, 1924; 203: 37–8.

[85] Bakradze TL (1974). Special features of the epidemiology of malaria in the process of its elimination in the Georgian SSR.

[86] Hay SI, Noor AM, Simba M, Busolo M, Guyatt HL, Ochola SA (2002). Clinical epidemiology of malaria in the highlands of western Kenya. Emerg Infect Dis.

[87] Sergiev VP, Baranova AM, Orlov VS, Mihajlov LG, Kouznetsov RL, Neujmin NI (1993). Importation of malaria into the USSR from Afghanistan, 1981–1989. Bull World Health Organ.

[88] Grumm RH (2011). The central European and Russian heat event of July–August 2010. Bull Am Meteorol Soc.

